# Gut Microbiota and Host Reaction in Liver Diseases

**DOI:** 10.3390/microorganisms3040759

**Published:** 2015-10-28

**Authors:** Hiroshi Fukui

**Affiliations:** Department of Gastroenterology, Endocrinology and Metabolism, Nara Medical University, 840 Shijo-cho Kashihara, 634-8522 Nara, Japan; E-Mail: hfukui@naramed-u.ac.jp; Tel.: +81-744-22-3051; Fax: +81-744-24-7122

**Keywords:** gut microbiota, endotoxin, alcoholic liver disease, nonalcoholic fatty liver disease, nonalcoholic steatohepatitis, liver cirrhosis, bacterial overgrowth, dysbiosis, bacterial translocation, bile acids

## Abstract

Although alcohol feeding produces evident intestinal microbial changes in animals, only some alcoholics show evident intestinal dysbiosis, a decrease in *Bacteroidetes* and an increase in *Proteobacteria*. Gut dysbiosis is related to intestinal hyperpermeability and endotoxemia in alcoholic patients. Alcoholics further exhibit reduced numbers of the beneficial *Lactobacillus* and *Bifidobacterium*. Large amounts of endotoxins translocated from the gut strongly activate Toll-like receptor 4 in the liver and play an important role in the progression of alcoholic liver disease (ALD), especially in severe alcoholic liver injury. Gut microbiota and bacterial endotoxins are further involved in some of the mechanisms of nonalcoholic fatty liver disease (NAFLD) and its progression to nonalcoholic steatohepatitis (NASH). There is experimental evidence that a high-fat diet causes characteristic dysbiosis of NAFLD, with a decrease in *Bacteroidetes* and increases in *Firmicutes* and *Proteobacteria*, and gut dysbiosis itself can induce hepatic steatosis and metabolic syndrome. Clinical data support the above dysbiosis, but the details are variable. Intestinal dysbiosis and endotoxemia greatly affect the cirrhotics in relation to major complications and prognosis. Metagenomic approaches to dysbiosis may be promising for the analysis of deranged host metabolism in NASH and cirrhosis. Management of dysbiosis may become a cornerstone for the future treatment of liver diseases.

## 1. Introduction

Human microbiota is estimated to contain as many as 10^14^ bacterial cells, which is a number 10 times greater than the human cells present in our bodies [1,2]. Above all, the gastrointestinal tract is the most heavily colonized organ for these microorganisms [1,2]. The human gut microbiota is dominated by the *Bacteroidetes* and the *Firmicutes*, whereas *Proteobacteria*, *Verrucomicrobia*, *Actinobacteria*, *Fusobacteria*, and *Cyanobacteria* are present in minor proportions [2,3]. The number of bacterial cells present in the mammalian gut increases toward the anal direction, *i.e.*, 10^1^~10^3^ bacteria per gram of contents in the stomach and duodenum, 10^4^~10^7^ bacteria per gram in the jejunum and ileum, and 10^11^~10^12^ cells per gram in the colon [2]. Samples from the small intestine were enriched for the *Bacilli* class of the *Firmicutes* and *Actinobacteria*, while *Bacteroidetes* and the *Lachnospiraceae* family of the *Firmicutes* were more prevalent in colonic samples [2,4]. The genetic information contained by the myriad of gut microbes encodes for a far more versatile metabolome than that found in the human genome [2,5]. Our own contribution to many of the metabolic processes essential for our homeostasis is remarkably small compared with the share provided by the microbiota [2]. The microbiota contributes to digestion, synthesis of vitamins, and resistance to intestinal colonization by pathogens, but also contains potentially pathogenic bacteria [6]. Disruption of intestinal homeostasis and alterations in the intestinal microbiome contribute to the pathogenesis of liver disease [6]. Intestinal bacterial overgrowth, changes in intestinal microbiota and the translocation of bacteria and their products are important common pathways for the development and progression of alcoholic liver disease, nonalcoholic fatty liver disease and liver cirrhosis. Their great impacts on the host lead to variable host reactions, which act bidirectionally to maintain the homeostasis on one side and to promote the liver disease on the other side.

Here, I would like to overview the roles of gut microbiota and host reaction in the process of alcoholic liver disease, nonalcoholic steatohepatitis, and liver cirrhosis.

## 2. Alcoholic Liver Diseases

Alcoholic liver disease (ALD) is a major cause of chronic liver disease worldwide and encompasses fatty liver (hepatic steatosis) and the more serious entities alcoholic steatohepatitis, alcoholic hepatitis, fibrosis, cirrhosis, and liver cancer [7,8]. In case of continued consumption of alcohol, fatty liver can progress to fibrosis and cirrhosis, which finally lead to portal hypertension and liver failure [7,8]. Alcohol abuse induces changes in the composition of gut microbiota and the translocation of bacterial products into the portal blood (bacterial translocation) appears to play a key role in alcohol-induced liver damage [9]. Chronic alcohol ingestion leads to small and large intestinal bacterial overgrowth and dysbiosis in animals and humans [8,10,11,12,13].

### 2.1. Alcohol-Related Dysbiosis

To date, there are few papers published on the effect of alcohol on the fecal microbiome. The experimental results obtained are not constant and probably depend on the different methods of alcohol feeding. Changes in intestinal microbiota associated with experimental and clinical studies on ALD are summarized in Table 1. After the intragastric feeding of alcohol (Tsukamoto-French method) to mice for three weeks, Yan *et al.* [13] reported a relative decrease of *Firmicutes* (*Lactococcus*, *Pediococcus*, *Lactobacillus*, and the *Leuconostoc* genera) and a relative increase of *Verrucomicrobia* and *Bacteroidetes* (*Bacteroidales*, *Bacteroides*, and *Porphyromonadaceae*). They [13] further found that *Lactobacillus* was strongly suppressed and almost absent in mice fed alcohol and reported that this finding may provide a rationale for the beneficial effect of various probiotic *Lactobacillus* strains. On the other hand, Bull-Otterson *et al.* [14] reported that the abundance of *Bacteriodetes* was rather decreased together with *Firmicutes* phyla, while those of *Proteobacteria* and *Actinobacteria* phyla were increased by the chronic *ad libitum* ethanol feeding with the Lieber-DeCarli ethanol liquid diet for eight weeks. In their study, the bacterial genera that showed the biggest expansion were the gram-negative alkaline-tolerant *Alcaligenes* and gram-positive *Corynebacterium* [14]. *Proteobacteria* includes several pathogenic species such as *Salmonella*, *Helicobacter*, *Vibrio* and *Escherichia*, one of the main bacteria in the gut [15]. Opportunistic infections of *Corynebacterium* members in patients with ALD were previously described in the USA and Sweden [16,17]. Three outbreaks of *Corynebacterium diphtheriae* infection in Seattle’s Skid Road from 1972 through 1982 were considered to be related to poor hygiene, crowding, season, and the contaminated fomites of urban alcoholic persons [16]. An outbreak of diphtheria during 1984 to 1986 in Sweden included 13 clinical cases and 65 carriers [17]. These reports suggested that *Corynebacterium diphtheria* may commonly overgrow in patients chronically exposed to alcohol [14].

Canesso *et al.* [18] further observed that the administration of ethanol in the drinking water for seven days to mice increased the colony-forming units (CFU) of *Enterobacteriaceae* in the contents of the small intestines. This *Enterobacteriaceae* group including *Escherichia coli* is associated with several pathological conditions in the gastrointestinal tract, such as inflammatory bowel disease [18].

Aerobic and anaerobic bacterial cultures of jejunal aspirates disclosed bacterial overgrowth in patients with chronic alcohol abuse [6,10]. Kirpich *et al.* [19] compared fecal microbiota of 66 alcoholic patients with 24 healthy controls. Their quantitative culturing of stool samples revealed that alcoholic patients had significantly reduced numbers of *Bifidobacteria*, *Lactobacilli*, and *Enterococci* [19]. Mutlu *et al.* [20] analyzed colonic biopsy samples by the 16S rRNA gene pyroseqencing and reported that the mean abundance of *Bacteroidaceae* from *Bacteroidetes* was significantly decreased in the alcoholic groups compared with the healthy controls. They found dysbiosis in 11 of 41 alcoholics. The alcoholics with dysbiosis had lower median abundances of *Bacteroidetes* and higher abundances of *Proteobacteria* (a uniform reduction of *Bacteroidetes*, decreases in *Clostridia* and increases in *Bacilli* and γ-*proteobacteria*) than alcoholics without dysbiosis. In their study, no correlation was noted between the duration of sobriety in sober alcoholics (defined as no alcohol consumption for at least one month) and the presence of dysbiosis, suggesting that the effects of chronic alcohol consumption on microbiota are long-lasting [20].

**Table 1:** Changes in intestinal microbiota associated with experimental and clinical studies on alcoholic liver disease (ALD).

Phylum	Class	Order	Family	Genus
*Firmicutes* ↓ ^[13,14]^	*Bacilli* ↑ ^[20]^*	*Bacillales*	*Bacillaceae*	*Bacillus* ↑ ^[20]*^
		*Lactobacilales*	*Lactobacillaceae*	*Lactobacillus* ↓ ^[13]^ **↓ ^[19]^**
				*Pediococcus* ↓ ^[13]^
			*Streptococcaceae*	*Lactococcus* ↓ ^[13]^
			*Lachnospiraceae*	*Blautia* ↑ ^[13]^
			*Leuconostocaceae*	*Leuconostoc* ↓ ^[13]^
			*Enterococcaceae*	*Enterococcus* **↓ ^[19]^**
	*Clostridia* ↓ ^[20]*,[21]†^	*Clostridiales*	*Clostridiaceae*	*Clostridium*
			*Ruminococcaceae* **↓ ^[21]†^**	*Ruminococcus* **↓ ^[21]†^**
				*Faecalibacterium* **↓ ^[21]†^**
				*Anaerofilum* **↓ ^[21]†^**
			*Oscillospiraceae*	*Oscillibacter* **↓ ^[21]†^**
			*Lachnospiraceae*	*Dorea* **↑ ^[21]†^**
				*Blautia* **↑ ^[21]†^**
	*Negativicutes*	*Selenomonadales*	*Acidaminococcaceae*	*Megasphaera* **↑ ^[21]†^**
*Actinobacteria* ↑ ^[14]^	*Actinobacteriia*	*Corynebactales*	*Corynebactaceae*	*Corynebacterium* ↑ ^[14]^
		*Bifidobacteriales*	*Bifidobacteriaceae*	*Bifidobacterium* **↓ ^[19,21]^**
*Verrucomicrobia* ↑ ^[13]^				
*Bacteroidetes* ↑ ^[13]^ ↓ ^[14]^ ↓ ^[20]*^	*Bacteroidia*	*Bacteroidales* ↑ ^[13]^	*Bacteroidaceae* **↓ ^[20]^**	*Bacteroides* ↑ ^[13]^
			*Porphyromonadaceae* ↑ ^[13]^	
*Proteobacteria* ↑ ^[14],^ ↑ ^[20]*^	*β-proteobacteria*	*Burkholderiales*	*Alcaligenaceae*	*Alcaligenes* ↑ ^[14]^
	*γ-proteobacteria* ↑ ^[20]*^	*Enterobacteriales*	*Enterobacteriaceae* ↑ ^[18]^	

Clinical data are displayed in blue; “↑” means increased abundance of bacteria compared with controls; “↓” means abundance of bacteria in patients compared with controls; Superior numbers are related reference numbers; ^[13]^ Yan (2011) mice, intragastric feeding of alcohol for three weeks; ^[14]^ Bull-Otterson (2013) mice, chronic *ad libitum* ethanol feeding by liquid diet for eight weeks; ^[18]^ Canesso (2014) mice, *ad libitum* ethanol feeding in the drinking water for seven days; ^[19]^ Kirpich (2008) alcoholic patients; ^[20]^ Mutlu (2012) alcoholic patients; ^[20]^*: alcoholics with dysbiosis compared to those without dysbiosis; ^[21]^ Leclercq (2014) alcoholic dependent patients with fibrosis stage F0~F1; ^[21]†^ alcoholics with dysbiosis compared to normal subjects.

Leclercq *et al.* [21] found that 26 of 60 (43%) liver fibrosis stage F0~F1 patients with alcohol dependence had elevated intestinal permeability (IP) by the ^51^Cr-ethylenediaminetetraacetic acid (EDTA) method. Interestingly enough, only patients with increased IP showed an altered fecal microbiota composition compared with control subjects. In alcohol-dependent subjects with high IP, bacteria from *Ruminococcaceae* and *Incertae Sedis XIII* were less abundant whereas those from *Lachnospiraceae* and *Incertae Sedis XIV* were more abundant compared with alcohol-dependent subjects with low IP and controls [21]. At the genus level of the bacterial groups, alcohol-dependent subjects with high IP had a drastic decrease in the abundance of *Ruminococcus*, *Faecalibacterium*, *Subdoligranulum*, *Oscillibacter*, and *Anaerofilum* belonging to the *Ruminococcaceae* family. The abundance of *Dorea* belonging to the *Lachnospiraceae* family was increased in alcohol-dependent subjects with high IP [21]. Additionally, the genera *Blautia* and *Megasphaera* were increased whereas *Clostridia* was decreased in alcohol-dependent subjects with high IP [21]. Their analysis further revealed that the total amount of bacteria and those belonging to the *Ruminococcaceae* family, especially *Faecalibacterium prausnitzii*, were negatively correlated to IP, while the genera *Dorea* and *Blautia* were positively correlated with IP. *F. prausnitzii*, which is also depleted in Crohn’s disease [22] and ulcerative colitis [23], has anti-inflammatory properties [22] and seems to be crucial for gut homeostasis [21]. They found a significant negative correlation between the abundance of *F. prausnitzii* and plasma Interleukin (IL)-8 and thought that alterations in microbial composition are associated with increased IP and increased plasma levels of proinflammatory cytokines [21]. The increases of *Lactobacillus* spp., *Bifidobacterium* spp. and the family *Ruminococcaceae* during alcohol abstinence suggest that these bacteria have a beneficial impact on gut-barrier function [24] and could contribute to the recovery of IP [21]. The effects of drinking on microbiota were analyzed in subjects with comparable body mass index (BMI) in the recent two studies [20,21].

In the fecal microbiom analysis of HBV- and alcohol-related cirrhotic patients, *Prevotellaceae* seem to be more represented in alcohol-related cirrhosis, although *Fusobacteria* were commonly increased in cirrhosis compared with controls [25]. They speculated the increase of *Prevotellaceae* is related to the ethanol metabolism in the human gut [25]. Kakiyama *et al.* [26] reported a decrease in two *Bacteroidetes* taxa, *Bacteroidaceae* and *Porphyromonadaceae*, and significant increase in a taxon from phylum *Firmicutes*, *Veillonellaceae*, in drinkers compared with nonalcoholic cirrhosis. *Veillonellaceae* specifically is linked with higher systemic inflammation and endotoxemia in cirrhotics [26].

A number of factors including intestinal dysmotility, increased gastric pH, and altered immune responses in addition to environmental and genetic factors might contribute to alcohol-associated dysbiotic changes [8]. Yan *et al.* [13] further proposed depressed antimicrobial activity of mucosal Reg3b and Reg3g (secreted c-type lectins) by alcohol as an additional factor for bacterial growth. MyD88-dependent Reg3g shows a potent bactericidal activity in intestinal epithelial and Paneth cells [27,28]. TLR2-dependent Reg3b expression in Peyer’s patches is an essential component in conditioning epithelial defense signaling pathways against bacterial invasion [29]. The lowest levels of Reg3b and Reg3g were observed in the proximal small intestine, where the bacterial overgrowth was most pronounced and luminal alcohol concentrations are highest [13]. Although many discrepancies to be explained exist in the details of intestinal microbial changes, it may be said that alcoholics exhibit reduced numbers of the beneficial *Lactobacillus* spp. [19] and, similarly to cirrhotics, show lower fecal amounts of *Bifidobacterium* spp. [8,19,21,30].

### 2.2. Intestinal Hyperpermeability and Bacterial Translocation

Bjarnason *et al.* [31] evaluated intestinal permeability by way of a ^51^Cr-EDTA absorption test and reported that non-cirrhotic alcoholic patients abstaining from alcohol for less than four days almost invariably had higher intestinal permeability than controls, and in many patients the abnormality persisted for up to two weeks after cessation of drinking. Keshavarzian *et al.* [32] reported that alcoholics with no liver disease showed a small but significant increase of intestinal permeability, but alcoholics with chronic liver disease demonstrated a marked and highly significant increase in intestinal permeability, estimated by a sugar test (urinary lactulose/mannitol ratio).

The passage of viable bacteria from the intestinal lumen through the intestinal wall and to the mesenteric lymph nodes and other sites are defined as bacterial translocation (BT). The concept of BT was later broadened to microbial products or their fragments, such as endotoxin (lipopolysaccharide (LPS)), peptidoglycan, lipopeptides, and bacterial DNA. The liver receives portal blood containing these microbial products and acts as the initial site of their filtration and detoxication [33]. BT is initiated when the intestinal epithelium is damaged and the intestine becomes more permeable [8,34,35]. Alcohol is known to disrupt gastrointestinal epithelial barrier integrity [36], resulting in the translocation of potentially harmful bacteria and their products such as endotoxins [10,37]. Alcohol and its metabolites, acetaldehyde and fatty acid ethyl esters, may contribute to the disruption of tight junctions, mainly through nitric oxide-mediated oxidative tissue damage and alterations in the cytoskeleton, but also through direct cell damage [38,39,40]. Alcohol-induced intestinal dysbiosis may be associated with intestinal epithelial barrier dysfunction [41].

### 2.3. Endotoxemia and Its Consequences

The best method to assess increased intestinal permeability is the direct measurement of bacterial products that originate only from the intestinal lumen, and must therefore have translocated into the extra-intestinal space, blood, and organs [6]. In 1991, we reported that irrespective of the stage of liver disease, alcohol abuse causes endotoxemia, not only in those with advanced cirrhosis but also in those with fatty liver [42]. There is strong evidence in support of the concept that gut-derived endotoxin as a marker for BT plays a central role in the initiation and progression of alcohol-induced liver injury [13]. Endotoxin is a major component of the gram-negative bacterial wall and acts as one of the pathogen-associated molecular patterns (PAMPs) for Toll-like receptors (TLRs). Once it reaches various organs, it powerfully stimulates TLR4 both in hepatic (Kupffer cells) and extrahepatic macrophages, which activates downstream signaling pathways responsible for overproduction of inflammatory cytokines such as TNF-α, IL-6, and IL-8. In addition, it is postulated that TLR4 and gut microflora-derived LPS contribute to the progression of liver fibrosis [43]. Alcohol induces lipopolysaccharide binding protein (LBP) and TLR4, and increases responsiveness to gut-derived endotoxin. The binding of LPS to CD14/TLR4 on Kupffer cells (KCs) activates production of cytokines and oxidants, which leads to T cell recruitment, hepatic stellate cell activation, and collagen production in the liver of patients with alcoholic steatohepatitis [33,44]. It should be noted that hepatic non-immune cells, such as hepatic stellate cell and endothelial cells, also respond to bacterial products through TLRs [45].

Lower median abundances of *Bacteroidetes* and higher ones of *Proteobacteria* in the colonic microbiome were proved to be correlated with high levels of serum endotoxin in alcoholics [20]. Endotoxemia and hepatic inflammation are considered as consequences of the expansion of the gram-negative bacteria from the *Proteobacteria* phylum, which occur in response to chronic ethanol consumption and can be prevented by the probiotic *Lactobacillus rhamnosus GG* [14]. A decrease in commensal probiotics could contribute to loss of the protective tight junction barrier [46]. This probiotic treatment also improved the decrease in tight junction protein expression [14].

The pathophysiological role of endotoxemia is further important in patients with severe alcoholic liver injury. Plasma endotoxin levels were increased with the progression of alcoholic liver injury and reached the maximal value in patients with alcoholic cirrhosis and severe alcoholic hepatitis who showed marked hypercytokinemia in our study [47]. Our animal experiments further revealed that endotoxemia and its effects on extrahepatic macrophages may play key roles in the progression of severe alcoholic liver injury and multiple organ failure [48].

Finally, studies using the direct measurement of microbiota function such as metagenomic, transcriptomic, and metabolomic assays (*i.e.*, the metabiome) are needed to determine whether changes in bacterial function rather than composition are important for the evaluation of host reaction in alcoholic liver diseases [20].

## 3. Nonalcoholic Steatohepatitis (NASH)

Nonalcoholic fatty liver disease (NAFLD) is the hepatic manifestation of the metabolic syndrome. It includes a spectrum of pathological changes ranging from the simple accumulation of fat in the liver through nonalcoholic steatohepatitis (NASH) to fibrosis, cirrhosis, and even hepatocellular carcinoma [49]. In addition, data show that NAFLD correlates with increased cardiovascular risk assessment scores and most of the clinical surrogates of cardiovascular diseases [50]. A link between inflammation and hepatic steatosis is shown both in alcoholic liver diseases and NAFLD [51,52,53]. An increased consumption of fructose may result in an increased lipid accumulation in the liver, which was accompanied by insulin resistance and elevated plasma triglycerides [53]. There is an accumulating evidence for the involvement of gut microbiota and bacterial endotoxin in some of the mechanisms of hepatic steatosis and its progression to NASH.

### 3.1. Obesity, NASH and Dysbiosis

Bacterial overgrowth, immune dysfunction, alteration of the luminal factors, and altered intestinal permeability are all involved in the pathogenesis of NASH and its complications [53]. Small intestinal bacterial overgrowth (SIBO) is a condition in which colonic bacteria translocate into the small bowel due to impaired microvilli function, which causes a breakdown in intestinal motility and gut homeostasis [2,54]. Most controlled trials demonstrated higher prevalence of SIBO (50%~77.8% *vs.* 9.1%~31.2%) in NAFLD patients compared with healthy subjects [49,55,56,57,58,59]. In contrast to these data obtained by breath tests, total bacterial counts in the feces, based on real-time PCR, did not differ between healthy subjects and persons with NAFLD or NASH [60]. Further studies are needed to determine whether fecal bacterial counts actually correlate with the amount of microbes present in the small intestine [6].

Ley *et al.* [61] first reported in their preliminary study that 12 obese patients were found to have higher abundance of *Firmicutes* and lower abundance of *Bacteroidetes* than did lean controls. Many efforts and much progress in experimental studies have been made after that in the research field of obesity and gut microbiota. In the obese mice, it has been established that the proportion of *Bacteroidetes* is decreased and that of *Firmicutes* is increased, therefore the *Firmicutes/Bacteroidetes* ratio is increased relative to their lean counterparts [62]. Turnbaugh *et al.* [63] added metagenomic and biochemical analysis in an experiment using *ob/ob* obese mice and showed that these microbial changes affect the metabolic potential of the mouse gut microbiota in the way that the obese microbiome has an increased capacity to harvest energy from the diet. They further found that microbiota transplantation from obese mice fed a high-fat diet to lean germ-free recipients promoted greater fat deposition than recipients from lean donors [64]. Hildebrandt *et al.* [65] investigated the microbial communities associated with switching from a standard chow diet to a high-fat diet in wild-type and resistin-like molecule (RELM) β knockout (KO) mice. The RELMβ gene is expressed by colonic goblet cells and its expression has been shown to be dependent on the gut microbiome [66] and can be induced by a high-fat diet [62,67]. These RELMβ KO mice remain comparatively lean on a high-fat diet. As a result, large alterations including a decrease in *Bacteroidetes* and an increase in both *Firmicutes* and *Proteobacteria* were noted for both genotypes, which suggested that the high-fat diet itself, and not the obese state, mainly accounted for the observed changes in the gut microbiota [65]. Turnbaugh *et al.* [68] further established the human gut ecosystem in mice by transplanting human fecal microbial communities into germ-free mice and reported that the high-fat, high-sugar “Western” diet shifted the structure of the microbiota, changed the representation of metabolic pathways in the microbiome, and altered microbiome gene expression. These mice had increased adiposity and their microbiota showed an increase in the representation of the *Erysipelotrichi* class of bacteria and the *Bacilli* (mainly *Enterococcus*) within the *Firmicutes* phylum as well as a decrease in the proportional representation of members of the *Bacteroidetes* [68].

Changes in intestinal microbiota associated with clinical studies on obesity are summarized in Table 2. Bacterial patterns are different between obese humans and lean humans; increased *Firmicutes* and decreased *Bacteroidetes* has been considered to be one identified pattern since the first report by Ley *et al.* [50,61]. However, the following clinical investigations have given variable and sometimes contradictory results [69,70,71,72,73,74,75,76,77]. In fact, several studies [72,73,74] reported an increased *Bacteridetes/Firmicutes* ratio in obese subjects.

Changes in intestinal microbiota associated with clinical studies on ΝΑFLD and NASH are summarized in Table 3. Recently, the fecal microbiota in NAFLD and NASH patients has been assessed using culture-independent techniques such as quantitative PCR and deep sequencing of a conserved region in the bacterial 16S rRNA gene [60,72,78,79]. Wong *et al.* [79] reported that NASH patients had lower fecal abundance of *Faecalibacterium* and *Anaerosporobacter* but higher abundance of *Parabacteroides* and *Allisonella*. The order *Aeromonadales*, the families *Succinivibrionaceae* and *Porphyromonadaceae*, and the genera *Parabacteroides* and *Allisonella* were more abundant in NASH patients than controls [79]. On the other hand, the class *Clostridia*, the order *Clostridiales*, and the genera *Faecalibacterium* and *Anaerosporobacter* were less abundant in NASH patients [79]. As written in the section of alcoholic liver disease, *Faecalibacterium prausnitzii* is an anti-inflammatory commensal which stimulates IL-10 secretion and inhibits IL-12 and interferon-γ expression [22]. The abundance of *Firmicutes* was unexpectedly lower in NASH patients than controls. It should be highlighted that like all human association studies, a causal relationship cannot be firmly established despite a trend toward a dose-dependent relationship [79]. Raman *et al.* [78] found over-representation of *Lactobacillus* species and selected members of phylum *Firmicutes* (*Lachnospiraceae*; genera, *Dorea*, *Robinsoniella*, and *Roseburia*) in the fecal microbiome of NAFLD patients, which was statistically significant. One member of phylum *Firmicutes* was significantly under-represented in NAFLD patients (*Ruminococcaceae*; genus, *Oscillibacter*) [78]. Zhu *et al.* [72] reported that *Proteobacteria*, *Enterobacteriaceae*, and *Escherichia* were the only phylum, family, and genus types exhibiting significantly increased levels in microbiomes of young NASH patients compared with obese children. They related increased abundance of *Escherichia* in stool microbiomes to elevation of blood alcohol in young NASH patients and led the attractive hypothesis (the alcohol hypothesis) that gut microbiota enriched in alcohol-producing bacteria (e.g., *E. coli*) constantly produce more alcohol than healthy microbiota and supply a constant source of reactive oxygen species to the liver, which, in turn, causes liver inflammation.

**Table 2:** Changes in intestinal microbiota associated with clinical studies on obesity.

Phylum	Class	Order	Family	Genus
*Firmicutes* ↑ ^[61]^ ↓ ^[72,73,74]^	*Bacilli*	*Bacillales*	*Stphylococcaceae*	*Staphylococcus* ↑ ^[71,76]^
		*Lactobacilales*	*Lactobacillaceae*	*Lactobacillus* ↑ ^[70], [77]†^
	*Clostridia*	*Clostridiales*	*Clostridiaceae*	*Clostridium* ↓ ^[74]^*
			*Eubacteriaceae* ↑ ^[71]^	*Eubacterium* ↓ ^[72]^
			*Ruminococcaceae* ↓ ^[72]^	*Ruminococcus* ↓ ^[72], [74]^*
				*Faecalibacterium* ↓ ^[72]^
			*Lachnospiraceae* ↓^ [72]^	*Roseburia* ↓^ [72]^
				*Blautia* ↓^ [72]^
				*Coprococcus* ↓^ [72]^
				*Lachnospira* ↓ ^[75]^
*Actinobacteria* ↑ ^[69]^	*Actinobacteriia*	*Bifidobacteriales*	*Bifidobacteriaceae*	*Bifidobacterium* ↓ ^[71,72,74]^
*Bacteroidetes* ↓ ^[61,69,70,71]^ ↑ ^[72,73,74]^	*Bacteroidia*	*Bacteroidales*	*Bacteroidaceae*	*Bacteroides* ↑ ^[76]^
			*Prevotellaceae* ↑ ^[72,75]^	*Prevotella* ↑ ^[72]^
*Proteobacteria* ↑ ^[72,73]^	*γ-proteobacteria*	*Enterobacteriales*	*Enterobacteriaceae*	*Eschericia (coli)* ↑ ^[71]^

“↑” means increased abundance of bacteria in patients *vs.* normal subjects; “↓” means decreased abundance of bacteria in patients *vs.* normal subjects; Superior numbers are related reference numbers; ^[61]^ Ley (2006); ^[69]^ Turnbaugh (2009); ^[70]^ Armougom (2009); ^[71]^ Santacruz (2010) preganant women; ^[72]^ Zhu (2013); ^[73]^ Yuan (2014); ^[74]^ Schwiertz (2010); ^[74]^* *C. leptum* group and *R. flavipaciens* subgroup; ^[75]^ Zhang (2009); ^[76]^ Collado (2008) pregnant women; ^[77]^ Million (2012); ^[77]† ^*L. reuteri*.

**Table 3:** Changes in intestinal microbiota associated with clinical studies on ΝΑFLD (in blue) and NASH.

Phylum	Class	Order	Family	Genus
*Firmicutes* **↑ ^[78]^** ↓ ^[79]^	*Bacilli*	*Lactobacilales*	*Lactobacillaceae*	*Lactobacillus* **↑ ^[78]^**
	*Clostridia* ↓^ [79]^	*Clostridiales* ↓ [79]	*Clostridiaceae*	*Anaerosporobacter* ↓ ^[79]^
			*Ruminococcaceae*	*Faecalibacterium* ↓ [79]
			*Lachnospiraceae* **↑ ^[78]^**	*Dorea* **↑ ^[78]^**
				*Roseburia* **↑ ^[78]^**
				*Robinsoniella* **↑ ^[78]^**
			*Oscillospiraceae*	*Oscillibacter* **↓ ^[78]^**
	*Negativicutes*	*Selenomonadales*	*Veillonellaceae*	*Allisonella* ↑ ^[79]^
*Bacteroidetes* ↓ ^[60]^	*Bacteroidia*	*Bacteroidales*	*Porphyromonadaceae* ↑ ^[79]^	*Parabacteroides* ↑ ^[79]^
*Proteobacteria* ↑ ^[72]^	*γ-proteobacteria*	*Aeromonadales* ↑ ^[79]^	*Succinivibrionaceae* ↑ ^[79]^	
	*γ-proteobacteria*	*Enterobacteriales*	*Enterobacteriaceae* ↑ ^[72]^	*Escherichia* ↑ ^[72]^

^[60]^ Mouzaki (2013) NASH patients compared to both simple steatosis and healthy controls; ^[78]^ Raman (2013) NAFLD patients without liver histology (probably including both hepatic steatosis and NASH); ^[72]^ Zhu (2013) young NASH patients compared with obese children; ^[79]^ Wong (2013) NASH patients.

Mouzaki *et al.* [60] reported that adult patients with NASH had a lower percentage of *Bacteroidetes* (*Bacteroidetes* to total bacteria counts) compared to both simple steatosis and healthy controls based on histological background. A lower percentage of *Bacteroidetes* could have affected energy balance by facilitating metabolic dominance of other bacteria that are more efficient in extracting energy from the diet. However, they did not find lower *Bifidobacteria* counts or a higher *Firmicutes/Bacteroidetes* ratio in NASH compared to simple steatosis and healthy controls [60]. This is in contrast to some of the previously published literature in the field of obesity [61,62] and the recent study by Zhu *et al.* on children with NASH [72].

Recently, Henao-Mejia *et al.* [80] experimentally disclosed that the nucleotide-binding domain, leucine-rich repeat protein (NLRP) 6 and NLRP3 inflammasomes and the effector protein IL-18 negatively regulate NAFLD/NASH progression via modulation of the gut microbiota. These inflammasomes are sensors of endogenous or exogenous pathogen-associated molecular patterns (PAMPs) or damage-associated molecular patterns (DAMPs) and regulators of the colonic microbiota [80]. Compared with wild-type mice, NLRP6 inflammasome-deficient mice exhibit both quantitative and qualitative changes in numerous taxa, including increased representation of members of *Prevotellaceae* and *TM7*, and reductions in members of genus *Lactobacillus* in the *Firmicutes* phylum [81]. Henao-Mejia *et al.* [80] showed that inflammasome deficiency-associated changes in gut microbiata are related to exacerbated hepatic steatosis and inflammation through influx of TLR4 and TLR9 agonists into the portal circulation in various experimental conditions. Co-housing of wild-type mice with these inflammasome-deficient mice resulted in exacerbations of hepatic steatosis, glucose intolerance, and obesity in wild-type mice. This observation may support the notion that gut dysbiosis itself can induce hepatic steatosis and metabolic syndrome [80].

Future studies should ideally include obese non-NAFLD patients or non-obese NAFLD patients to exclude the impact of obesity or control for obesity in statistical analysis [82]. It should be further noted that many studies so far excluded all taxa with an abundance below 1%. However, even low-abundance bacteria such as *Akkermansia muciniphila* have the potential to profoundly affect host metabolism [83]. This mucin-degrading bacterium in the mucus layer was reported to reverse high-fat diet-induced metabolic disorders, including fat-mass gain, metabolic endotoxemia, adipose tissue inflammation, and insulin resistance [84]. *A. muciniphila* also increases the intestinal levels of endocannabinoids that control inflammation, the gut barrier, and gut peptide secretion [84].

As stated above, the studies comparing the bacterial taxonomic composition of patients with NAFLD *vs.* those with NASH indicated variable and even contradictory findings [6]. Changes in bacterial taxonomy might not be as important as changes in bacterial genes (metagenomics and metatranscriptomics) in the development of NAFLD and NASH [6]. Obesity is accompanied by an intestinal metagenome that has an increased capacity to collect energy from the host diet. Bacterial enzymes aid in the digestion of otherwise indigestible dietary polysaccharides and the extraction of calories from them [6,63].

### 3.2. Metabolic Changes Related to Gut Microbiota

In addition to microbial cells or microbial structural components, microbial metabolites also have the ability to affect the health and disease of the host. Human colonic microbiota break down substrates such as resistant starch and nonstarch polysaccharides (major components of dietary fiber), which are not completely hydrolyzed by host enzymes in the small intestine [85]. The main fermentation products ensuing from this fiber breakdown are the short chain fatty acids (SCFAs) acetate, propionate, and butyrate [85], which can be utilized for lipid or glucose *de novo* synthesis [86]. It has been estimated that these SCFAs constitute 3%–9% of our daily caloric intake [87]. The bacterial SCFAs thus provide an additional source of energy for the body [74]. Increased production of SCFAs by the gut microbiota, first noted in *ob/ob* mice [63], was also observed in overweight and obese people compared to lean subjects [74]. Schwiertz *et al.* [74] found an increased total amount of fecal SCFA in obese subjects and increased propionate in overweight and obese subjects compared with lean subjects, which is in line with the thought that SCFA metabolism may play a considerable role in obesity. However, the fecal *Bacteridetes/Firmicutes* ratio and the proportion of *Bacteridetes* were increased in their study [74], which was in contrast to the previous reports [61,69,70] with regard to the contribution of bacterial groups to the development of obesity. Although members in both phyla produce SCFAs, it seems that *Bacteroidetes* (e.g., *Prevotella*) produce more SCFAs [88]. Zhu *et al.* [72] also reported that *Bacteroidetes* (mainly *Prevotella*) were significantly elevated in obese and NASH children compared to lean healthy children and that their cross-sectional study supported a causal relationship between high *Prevotella* and obesity. Although the meaning of dysbiosis is still controversial, the amount of SCFAs produced by intestinal microbiota seems to contribute to the development of obesity and obesity-related NAFLD.

It has been proposed from the study of inflammatory bowel diseases that the G protein-coupled receptor 43 (GPR43), a receptor for SCFAs, suppresses inflammatory responses in the gut [89]. GPR43, also termed free fatty acid receptor 2 (Ffar2), is expressed in the rat and human colon wall, in mucosa, and in enteroendocrine cells expressing peptide YY [90,91]. By suppressing experimental colitis, SCFAs/GPR43 signaling may help to improve gut permeability and therefore minimize the hepatic injuries imposed by microbial cell components and microbial products [89]. On the other hand, the fact that *Ffar2* (GPR43)-deficient (*Ffar2*-KO) mice are completely protected from high-fat diet-induced obesity, dyslipidemia, and fatty liver [92] supports the importance of SCFAs/GPR43 signaling in the development of NAFLD.

Bile acids (BAs) are saturated, hydroxylated C-24 cyclopentanophenanthrene sterols synthesized from cholesterol in hepatocytes [87]. Cholesterol 7α -hydroxylase (CYP7A1) produces both the dihydroxy BA chenodeoxycholic acid (CDCA) and the trihydroxy BA cholic acid (CA) in the liver. These BAs are conjugated to glycine or taurine before being secreted from the liver and stored in the gallbladder. Eating stimulates gallbladder contraction and emptying of the contents into the small intestine [93]. Bile salts solubilize fats and fat-soluble vitamins before they are absorbed in large part (~95%) by the ileum, and return to the liver by way of the portal vein, thus completing portal enterohepatic circulation (EHC). The rest escapes the EHC and becomes substrate for microbial biotransforming reactions in the colon [87]. The colonic 7α-dehydroxylating bacteria are known to convert primary BAs, CDCA, and CA, into secondary BAs lithocholic acid (LCA) and deoxycholic acid (DCA), respectively.

BAs are now recognized as signaling molecules capable of activating specific nuclear farnesoid X receptor (FXR) and membrane BA-activated G protein-coupled receptor (GP-BAR1) TGR5 [94,95,96,97]. CDCA is the most potent endogenous FXR ligand. Secondary BAs, DCA and LCA, can also activate to a much lesser extent. TGR5 is activated by nanomolar concentrations of LCA and micromolar concentrations of CA, DCA, and CDCA [41,98]. By activating various signaling pathways after binding to FXR mainly in the enterocytes and the parenchymal hepatocytes and to TGR5 especially in the non-parenchymal hepatocytes, BAs regulate various metabolic processes, including triglyceride, cholesterol, and glucose metabolism and inflammatory reactions [98]. Beneficial effects of BAs and agonists such as FXR and TGR5 ligands on lipid and glucose metabolism and liver pathology have been reported one after another [99,100,101,102,103,104,105,106,107,108]. Developing new strategies for the management of liver and associated metabolic disorders along these lines has been in the focus of research interest, before exploring the relationships of these BAs receptors to gut microbiota and the metabolic syndrome. In addition to other studies supporting the beneficial effect of FXR ligands on liver inflammation and fibrosis [100,101,102,104], the fact that FXR-deficient mice fed a methionine/choline-deficient diet (MCDD) developed more severe liver injury but a lower degree of steatosis suggests the role of BAs and FXR in maintaining liver homeostasis against metabolic syndrome. These interesting studies are, however, beyond the field of this review.

The effect of the gut microbiome on host metabolism is also exemplified by the relation of high-fat diets and choline deficiency. High-fat diet leads to the formation of intestinal microbiota that convert dietary choline into methylamines [109]. This microbiota-related reduced choline bioavailability may result in the inability to synthesize phosphatidylcholine, which is necessary for the assembly and secretion of very-low-density lipoprotein (VLDL) [110] and the subsequent accumulation of triglycerides in liver. The high-fat diet thus mimics the effect of choline-deficient diets, causing NAFLD [109].

### 3.3. Intestinal Permeability, Endotoxemia and Bacterial Translocation

Miele *et al.* [49] provided the first evidence of intestinal permeability and decreased tight junction protein zonula occludens-1 (ZO-1) expression in biopsy-proven NAFLD patients compared with healthy subjects. Intestinal permeability is increased in children with non-alcoholic fatty liver disease, and correlates with the severity of the disease [111].

Children with NAFLD had significantly higher serum concentrations of endotoxins than control subjects [112,113]. Patients with NASH revealed higher serum endotoxin levels and higher intensity of TLR4 protein expression in the liver compared to patients without NASH [114]. Hepatic TLR4 expression was further proved to be up-regulated in a large cohort of NASH patients when compared to those with NAFLD, and this occurs within the setting of increased plasma endotoxin and fatty acids [115]. An importance of TLR4 signaling was further suggested by the study reporting that TLR4 codon 299 heterozygous gene mutation (Asp299Gly) was significantly lower in the NAFLD than in the control group [116].

Experimentally, genetically obese *ob/ob* and *db/db* mice showed enhanced intestinal permeability, profoundly modified distribution of occludin and ZO-1 in the intestinal mucosa together with higher circulating levels of inflammatory cytokines, and portal endotoxemia compared with lean control mice [117]. Lastly, growing evidence suggests that at the early course of a high-fat diet, not only bacterial products but also complete living bacteria can be translocated from the intestinal lumen towards tissues, such as the adipose tissue [118,119], enhancing the role of BT and gut permeability in tissue injury [50].

The alcohol hypothesis of NASH could explain the similarity of liver histology observed in alcoholic steatohepatitis and NASH and may partly explain the observation of increased gut permeability [49] and endotoxemia in NASH patients [120], because alcohol is known to increase gut permeability [58]. The gene expression of all three major pathways for ethanol catabolism in NASH liver is proved to be highly elevated [121]. Recently, elevated blood ethanol concentration was observed in NAFLD patients [58]. Zhu *et al.* [72] additionally showed that the blood ethanol concentration of obese patients without NASH is not elevated; however, obese patients with NASH had significantly elevated blood ethanol.

### 3.4. NASH as a Derangement of Gut-Liver Axis

Mice deficient in PAMPs or downstream signaling are resistant to NASH [122,123]. Dietary habits, by increasing the percentage of intestinal gram-negative endotoxin producers, may accelerate liver fibrogenesis, introducing dysbiosis as a cofactor contributing to chronic liver injury in NAFLD patients [124]. The pathogenetic mechanism of NASH has been recently elucidated from the standpoint of the gut-liver axis. Rivera *et al.* [123] first reported the importance of TLR4 signaling in the liver. They observed histological evidence typical of steatohepatitis, portal endotoxemia, and enhanced TLR4 expression in wild-type mice fed MCDD. In contrast, injury and lipid accumulation markers were significantly lower in TLR4 mutant mice. The destruction of KCs with clodronate liposomes blunted histological evidence of steatohepatitis and prevented increases in TLR4 expression.

We have reported the enhanced expressions of TNF-α, TLR4, and CD14 mRNA and the reduced phagocytic function of the KCs in the rat NASH model fed a choline-deficient l-amino-acid-defined (CDAA) diet [125,126]. We further reported higher TNF-α production from isolated KCs and greater staining of TNF-α, TLR4, and macrophage/dendritic cells in the submucosa of ileum in this NASH model [127]. One unsolved problem for this endotoxin hypothesis is why KCs were activated by low-level endotoxin in the portal blood [128]. Imajo *et al.* [128] proposed an interesting notion that endotoxin hyper-responsivity related to enhanced CD14 expression in KCs is important for the progression of NASH. On the other side, another key point seems to be hidden in the KCs, which showed a dissociation between the phagocytic function and the proinflammatory function in our above experiments. Large amounts of microbial products together with lipids attributable to high-fat diet and dysbiosis in the intestine are considered to reach the liver via the portal circulation in patients with NASH. Phagocytic function of KCs is overwhelmed by taking these microbial products. However, the activation of TLR4 signaling may proceed without intracellular uptake of endotoxin, because TLR4 is the signaling but not the LPS uptake receptor [129]. Overwhelming endotoxin may activate TLR4 on the hepatic stellate cell, which enhances the production of extracellular matrix and liver fibrosis.

Recently, our group further reported enhanced α-SMA expression (suggesting hepatic stellate cell activation), elevated liver LBP mRNA levels (suggesting portal endotoxemia), increased intestinal permeability, and decreased intestinal tight junction protein expression in the above rat NASH model [130]. We also proved that inhibition of LPS-TLR4 signaling with oral administration of poorly absorbable antibiotics improved all of these intestinal and liver events and inhibited the progression of liver fibrosis [130].

The importance of the gut-liver axis and TLR4 signaling in the progression of NAFLD has been confirmed by other experimental studies as well. C3H/HeJ mice, which have a loss-of-function mutation in TLR4, are protected against the development of diet-induced obesity [131]. These mice demonstrate decreased adiposity, increased oxygen consumption, a decreased respiratory exchange ratio, improved insulin sensitivity, and enhanced insulin-signaling capacity in adipose tissue, muscle, and liver compared with control mice during high-fat feeding [131]. TLR4 and its coreceptor, myeloid differentiation factor-2 (MD-2), are keys in the recognition of LPS and the activation of proinflammatory pathways. In MD-2 KO and TLR4 KO mice fed MCDD, liver triglyceride accumulation and increased thiobarbituric acid reactive substances, a marker of lipid peroxidation, were significantly attenuated [132].

In addition to TLR4, TLR2 and TLR9 are considered to play a role in NAFLD pathogenesis [133,134]. TLR9 recognizes DNA containing an unmethylated-CpG motif, which is rich in bacterial DNA [135]. Bacterial DNA is detectable in the blood and liver. TLR9 expression is increased in experimental NASH models. [122,133]. TLR9-deficient mice on the CDAA diet showed less steatosis, inflammation, and liver fibrosis compared with their wild type (WT) counterparts [122]. TLR2 perceives components of gram-positive bacterial cell walls such as peptidoglycan and lipoteichoic acid [135]. TLR2-deficient mice are resistant to CDAA-induced steatohepatitis, showing lower expression of proinflammatory cytokines [136].

## 4. Liver Cirrhosis

Liver cirrhosis is a frequent consequence of the long clinical course of all chronic liver diseases and is characterized by tissue fibrosis and the conversion of normal liver architecture into structurally abnormal nodules [137]. Portal hypertension underlies most of the clinical complications of the disease [137]. Bacterial infections account for significant morbidity and mortality [138] and infections increase mortality four-fold in cirrhotic patients [139]. Although urinary, respiratory, ascitic fluid infections and bacteremia are common infectious complications, spontaneous bacterial peritonitis (SBP) occurs most frequently. A vast majority of such infections are due to enteric gram-negative bacteria, mainly *Enterobacteriaceae* [140]. The investigation of the gut microbiome in cirrhosis is important because of the key roles of BT and endotoxemia in the pathogenesis of various complications [141].

### 4.1. Small Intestinal Bacterial Overgrowth and Dysbiosis

Small intestinal bacterial overgrowth (SIBO) is a condition in which colonic bacteria translocate into the small bowel due to impaired microvilli function, which causes a breakdown in intestinal motility and gut homeostasis [2,54]. SIBO, defined as ≥10^5^ total colony-forming units per milliliter of proximal jejunal aspirations, was present in 59% of cirrhotic patients and is associated with systemic endotoxemia [142]. SIBO, also determined by the breath hydrogen test, is common in cirrhotics, especially in those with ascites and advanced liver dysfunction and in those with a history of SBP. Delayed gut transit may be associated with the development of SIBO [38]. Orocecal transit time (OCTT) and small-bowel residence time in cirrhotics with SIBO were significantly longer than in those without it [143,144]. Acceleration of orocecal transit by cisapride in animals and humans is reported to lead the abolishment of bacterial overgrowth in cirrhotic patients with bacterial overgrowth [145]. The exact etiology for delayed intestinal transit in patients with liver cirrhosis is largely unknown but could be multifactorial [143]. It could be due to the autonomic neuropathy, metabolic derangements, and diabetic state complicated in cirrhotic patients [143]. In addition, SIBO itself may lead to delayed intestinal transit in patients with cirrhosis [143], for antibiotic therapy has been shown to reduce the OCTT in cirrhotics [146]. 

Chen *et al.* [25] reported that the proportion of phylum *Bacteroidetes* was significantly reduced, whereas *Proteobacteria* and *Fusobacteria* were highly enriched in fecal microbiota of patients with hepatitis B virus (HBV)-related and alcoholic cirrhosis (24 HBV-related and 12 alcoholic) compared with healthy controls. This finding was further confirmed by their recent report on 123 cirrhotics compared with 114 healthy counterparts [147], which revealed a decrease of the genera *Bacteroides*, *Eubacterium*, and *Alistipes*, whereas *Veillonella*, *Streptococcus*, *Clostridium*, and *Prevotella* were increased compared to healthy controls [147].

Changes in intestinal microbiota associated with clinical studies on liver cirrhosis are summarized in Table 4. Bajaj *et al.* [148] reported that fecal microbiota in liver cirrhosis (52% alcoholic, 40% hepatitis C virus-related, and 8% cryptogenic) showed a lower proportion of *Ruminococcaceae* and *Lachnospiraceae* and a higher proportion of *Enterobacteriaceae*, *Alcaligeneceae*, and *Fusobacteriaceae* compared with healthy controls. Lu *et al.* [149] analyzed fecal microbiota of asymptomatic carriers of hepatitis B virus (HBV), chronic hepatitis B patients, and decompensated HBV-related cirrhotics compared with healthy controls, and HBV-related cirrhosis showed a significant decrease of *Faecalibacterium prausnitzii*, *Bifidobacteria*, and *Clostridium* and a significant increase of *Enterococcus faecalis* and *Enterobacteriaceae* compared with other groups [149]. They also showed that the *Bifidobacteria*/*Enterobacteriaceae* (B/E) ratio is decreased with the progression of liver diseases [149]. Xu *et al.* [150] reported that *Bifidobacterium*, especially *Bifidobacterium catenulatum*, was decreased in the stool of HBV-related cirrhotics compared with the controls. Furthermore, Wu *et al.* [151] noted marked decrease in the population of *Lactobacillus rhamnosus* and a reduction in the frequency of *Lactobacillus fermentus* in HBV-related decompensated cirrhosis. Analysis of rectosigmoidal mucosal microbiom by Bajaj *et al.* [152] revealed a significantly lower abundance of autochthonous genera (*Dorea*, *Subdoligranulum* and *Incertae Sedis XIV* other) and a higher abundance of potentially pathogenic ones (*Enterococcus*, *Proteus*, *Clostridium*, and *Burkholderia*) in cirrhotic patients compared with controls.

**Table 4:** Changes in intestinal microbiota associated with clinical studies on liver cirrhosis.

Phylum	Class	Order	Family	Genus
*Firmicutes*	*Bacilli*	*Bacillales*	*Stphylococcaceae*	*Staphylococcus* ↑^ [156]†^
		*Lactobacilales*	*Lactobacillaceae*	*Lactobacillus* ↓ ^[151]^
			*Streptococcaceae*	*Streptococcus* ↑ ^[147]^
			*Enterococcaceae*	*Enterococcus* ↑ ^[149,152]^
	*Clostridia*	*Clostridiales*	*Clostridiaceae*	*Clostridium* ↓ ^[149]^ ↑ ^[147,152]^
			*Eubacteriaceae*	*Eubacterium* ↓ ^[147]^
			*Ruminococcaceae* ↓ ^[148,157]^	*Subdoligranulum* ↓ ^[152]^
				*Faecalibacterium* ↓ ^[149]^
			*Lachnospiraceae* ↓ ^[148,157]^	*Dorea* ↓ ^[152]^
				*Blautia* ↓ ^[157]^
	*Negativicutes*	*Selenomonadales*	*Veillonellaceae*	*Veillonella* ↑ ^[147]^
			*Acidaminococcaceae*	*Acidaminococcus* ↑ ^[152]^
*Actinobacteria*	*Actinobacteriia*	*Bifidobacteriales*	*Bifidobacteriaceae*	*Bifidobacterium* ↓ ^[149,150]^
*Fusobacteria* ↑ ^[25,147]^	*Fusobacteriia*	*Fusobacteriales*	*Fusobacteriaceae* ↑ ^[148]^	
*Bacteroidetes* ↓ ^[25,147]^	*Bacteroidia*	*Bacteroidales*	*Bacteroidaceae*	*Bacteroides* ↓ ^[147]^
			*Prevotellaceae*	*Prevotella* ↑ ^[147]^
			*Rikenellaceae*	*Alistipes* ↓ ^[147]^
*Proteobacteria* ↑ ^[25,147]^	*β-proteobacteria*	*Burkholderiales*	*Alcaligenaceae* ↑^ [148]#^	
			*Burkholderiaceae*	*Burkholderia* ↑ ^[152]^
			*Ralstoniaceae*	*Ralstonia* ↑ ^[152]^
	*γ-proteobacteria*	*Enterobacteriales*	*Enterobacteriaceae* ↑ ^[25,148], [148]# [149,157]^	*Proteus* ↑ ^[152]^
				*Eschericia* (*coli*) ↑^ [156]†^

“↑” means increased abundance of bacteria in patients *vs.* normal subjects; “↓” means decreased abundance of bacteria in patients *vs.* normal subjects; Superior numbers are related reference numbers; ^[25]^ Chen (2011); ^[147]^ Qin (2014); ^[148]^ Bajaj (2012); ^[148]#^ means cirrhotics with hepatic encephalopathy (HE) *vs.* healthy controls and cirrhotic patients without HE; ^[149]^ Lu (2011); ^[150]^ Xu (2012); ^[151]^ Wu (2012); ^[152]^ Bajaj (2012b); ^[156]^ Liu (2004); ^[156]†^ means cirrhotics with minimal HE *vs.* healthy controls; ^[157]^ Kakiyama (2013).

A *post hoc* analysis of a comprehensive study of the fecal microbiome by Bajaj *et al.* [141] revealed that alcoholic cirrhotics had a significantly lower abundance of *Lachnospiraceae*, *Ruminococcaceae*, and *Clostridialies XIV* and a higher abundance of *Enterobacteriaceae* and *Halomonadaceae* despite a statistically similar Model for End-stage Liver Disease (MELD) score and BMI compared to those without alcoholic etiology. They further reported a lower abundance of *Veillonellaceae* and a higher abundance of *Porphyromonadaceae*, *Bacterioidaceae* in NASH cirrhotics than in the non-NASH counterparts [141].

The term “cirrhosis dysbiosis ratio”, or CDR, was proposed to reflect the changes of “good” *vs.* “bad” bacteria occurring in the intestine of cirrhotic patients [141]. This ratio consists of the amount of the beneficial autochthonous (*Lachnospiraceae* + *Ruminococaceae* + *Veillonellaceae* + *Clostridiales Incertae* *Sedis XIV*) and potentially pathogenic taxa abundance (*Enterobacteriaceae* + *Bacteroidaceae*). It is postulated that the lower the CDR, the more advanced is the cirrhosis, for there was a significant negative correlation of the CDR with MELD score and blood endotoxin level [141]. This reduction in autochthonous taxa can be disruptive for the cirrhotics because they produce SCFAs that reduce colonic inflammation, compete with pathogenic bacteria for nutrients, produce anti-bacterial peptides, and may improve the intestinal barrier [141,153,154]. The CDR is lowest in alcoholic cirrhotic patients compared with cirrhotic subjects of another etiology; similarly, endotoxemia is higher and correlates with the expanding gram-negative *Enterobacteriaceae* in these alcoholic patients [8,141].

Several studies show that gut microbiota is altered in cirrhotic patients with hepatic encephalopathy (HE) [155]. Liu *et al.* [156] reported the results in patients with cirrhosis mostly (70%–80%) related to hepatitis B virus (HBV) or hepatitis C virus (HCV) and found a significant fecal overgrowth of potentially pathogenic *Escherichia coli* (*E. coli*) and *Staphylococcus* spp. in the gut microbiota of cirrhotics with minimal HE. Bajaj *et al.* [148] showed that patients with cirrhosis and HE had a higher concentration of *Enterobacteriaceae* and *Alcaligenaceae* compared with control subjects [155]. In their cirrhosis group, *Alcaligeneceae* and *Porphyromonadaceae* were positively correlated with cognitive impairment. The association of *Alcaligeneceae* and HE may be partly explained by the fact that they degrade urea to produce ammonia [148]. They further studied colonic mucosal microbiota and found that cirrhotics with HE had lower *Roseburia* and higher potentially pathogenic genera *Enterococcus*, *Veillonella*, and *Burkholderia* compared to those without HE, although fecal microbiota was not different between the two groups [152].

### 4.2. Metabolic Changes Related to Gut Microbiota

Cirrhotics, compared to controls, had a higher *Enterobacteriaceae* (potentially pathogenic) but lower *Lachonospiraceae*, *Ruminococcaceae*, and *Blautia* (7α-dehydroxylating bacteria) abundance. The gut 7α-dehydroxylating bacteria are known to convert primary BAs, CDCA and CA, into secondary BAs, LCA and DCA, respectively. Kakiyama *et al.* [157] analyzed fecal microbiota and BAs and found that CDCA was positively correlated with *Enterobacteriaceae* and DCA was positively correlated with *Ruminococcaceae*. They further noted a positive correlation between *Ruminococcaceae* and DCA/CA and a positive correlation between *Blautia* and LCA/CDCA. These suggest that a decreased conversion of primary to secondary fecal BAs is linked with an abundance of key gut microbiome taxa in advanced liver cirrhosis. BT leads to inflammation, which suppresses synthesis of total BAs in the liver via the inhibition of CYP7A1. BAs prevent BT and avoid the passage of bacterial products from the lumen of the intestine [158,159,160]. A decrease in BAs entering the intestines appears to favor overgrowth of pathogenic and proinflammatory members of the microbiome including *Porphyromonadaceae* and *Enterobacteriaceae* [93]. Kakiyama *et al.* [26] further reported that actively drinking cirrhotics revealed enhanced expression of FXRα mRNA in the ileal and sigmoid colonic mucosa together with increased expression of TNF-α, IL-1β, IL-6 and MCP-1 mRNA in the colonic mucosa.

New strategies that specifically target BA receptors may open new doors in the treatment of liver cirrhosis as well. Renga *et al.* [161] reported that *in vivo* activation of FXR regulates the expression of genes involved in glutamine/glutamate metabolism and stimulates urea synthesis and ammonia detoxification in a rodent model of cirrhosis [161]. Verbeke *et al.* [162] showed that FXR agonist obeticholic acid improves portal hypertension in two different rat models of cirrhosis by decreasing intrahepatic vascular resistance by increasing intrahepatic eNOS activity. They further reported that this FXR agonist prevents gut barrier dysfunction, intestinal inflammation, and BT in cholestatic rats, and thus demonstrated a crucial protective role of FXR in the gut-liver axis [163].

The metagenomic approach to intestinal microbiota seems more important for understanding the pathophysiology of liver cirrhosis, where variable metabolic and immunologic changes play pivotal roles. Metagenomic pyrosequencing of intestinal microbiota has led to the discovery of novel genes from uncultivated microorganisms, and the assembly of whole genomes from community DNA sequence data [164]. Chen *et al.* [164] performed a functional gene array to study the gut microbiome in patients with alcoholic and HBV-related cirrhosis and healthy controls and found that the functional composition of fecal microbiomes was mostly influenced by alcohol consumption, and secondly by cirrhosis. Alcohol consumption caused significant enrichment of functional genes including xenobiotic metabolism and virulence, while both cirrhosis groups were markedly depleted in the functional genes involved in nutrient processing, such as amino acid metabolism, lipid metabolism, and nucleotide metabolism [164].

Qin *et al.* [147] grouped the microbial genes into clusters, and denoted metagenomic species (MGS) on the basis of their abundance profiles to explore further the microbial genes associated with liver cirrhosis. At the module or pathway level, the liver cirrhosis-associated markers included assimilation or dissimilation of nitrate to or from ammonia, denitrification, GABA (γ-aminobutyric acid) biosynthesis, GABA shunt, hem biosynthesis, phosphotransferase systems, and some types of membrane transport, such as amino acid transport. On the other hand, the most prevalent markers among the controls included those involved in carbohydrate metabolism, amino acid metabolism, energy metabolism, signal transduction, and the metabolism of cofactors and vitamins. The enrichment of the modules for ammonia production and GABA biosynthesis in cirrhotics suggests a potential role of gut microbiota in hepatic encephalopathy (HE) [147].

### 4.3. Intestinal Permeability, Endotoxemia and Bacterial Translocation

Structural and functional changes in the intestinal mucosa that increase the intestinal permeability of bacteria and its products have been found in patients with liver cirrhosis [158]. This intestinal barrier dysfunction has been regarded as an important pathogenetic factor for several complications of liver cirrhosis [38]. Alcohol drinking, portal hypertension, alterations in the intestinal microbiota, inflammation and oxidative stress, and endotoxemia can all affect barrier function of both the small and large intestine and may contribute to the development of cirrhotic complications [41]. Many authors [34,41,165,166,167] have reported that patients with liver cirrhosis revealed intestinal hyperpermeability, the state of so-called “leaky gut”. This intestinal hyperpermeability has been reported to be more common in cirrhotics with a history of SBP [167], associated with severity of liver cirrhosis as assessed by the Child-Pugh classification [165,166,167], and also has been considered as a predictor of bacterial infections [168]. Pijls *et al.* [169] showed that small intestinal permeability determined by urinary lactulose/rhamnose excretion ratio is not altered, whereas large intestinal permeability is increased in patients with stable compensated cirrhosis. As larger numbers and diversity of bacteria are present in the large intestine, an increased permeability of this site may enhance the risk of BT [169].

The passage of viable bacteria from the intestinal lumen through the intestinal wall and their translocation to mesenteric lymph nodes and other sites is the accepted pathogenic mechanism for the development of spontaneous infections, such as SBP or bacteremia [158]. Bacterial products, such as endotoxin or bacterial DNA can translocate to extra-intestinal sites and promote an immunological response similar to that produced by viable bacteria. Pathological BT is a contributing factor in the development of complications in cirrhosis, not only in infections, but by exerting a profound inflammatory state and exacerbating the hemodynamic derangement [41,158]. Gut flora imbalances, and higher levels of *Enterobacteriaceae* result in significant changes in BT and liver function in cirrhotic rats [170]. Further, SIBO in cirrhosis showed a high correlation with the presence of bacterial DNA fragments in peripheral blood, suggesting that SIBO could be a major risk factor of BT, especially in ascitic patients [171].

Currently, more than 10 members of the TLR family have been identified. TLR4 was the first identified isoform that responds primarily to LPS [45]. LPS binds to TLR4 with co-receptor CD14 and MD-2. TLR2 heterodimerizes with TLR1 or TLR6 to recognize lipoprotein and peptidoglycan derived from gram-positive bacteria. Bacterial flagellin is recognized by TLR5. Intracellular TLR3 and TLR9 are activated by microbe-derived nucleic acids including double-stranded RNA and CpG motif containing unmethylated DNA, respectively [43]. Plasma endotoxin assay has been most widely used as a reliable marker of BT, although many methodological controversies still exist [33].

The depressed elimination of endotoxin by KCs causes spillover endotoxemia. Decreased endotoxin inactivation in the blood is considered to enhance the processing of endotoxin by extrahepatic macrophages which secrete a larger amount of TNF than KCs [33,172,173]. The excessive cytokine response to endotoxin by splenic and alveolar macrophages may be important in the pathogenesis of acute respiratory distress syndrome (ARDS) and multiple organ failure in advanced liver cirrhosis [33,173]. Endotoxemia enhances vascular nitric oxide (NO) production, which is the primary stimulus for the development of systemic peripheral vasodilatation. Hyperdynamic circulation characterized by hypotension, low systemic vascular resistance, high cardiac output, and a reduced sensitivity to vasoconstrictors are features of cirrhosis. Cirrhotic patients showed significant increases in serum nitrite/nitrate which was significantly correlated with endotoxemia [174]. The oral administration of antibiotic colistin to cirrhotic patients reduced plasma endotoxin levels and serum nitrite/nitrate levels [174]. Enhanced vasoconstrictive factors in response to vasodilatation and endotoxemia are responsible for ascites and hepatorenal syndrome. HE is also closely related to inflammatory reaction attributable to leaky gut and endotoxemia [33]. Close relationships of endotoxemia to other various cirrhotic complications such as portal hypertension, renal disturbance, cardiomyopathy, hepatopulmonay syndrome, and coagulation and platelet abnormalities were summarized in my previous review [33].

LBP can be evaluated as another useful surrogate marker of BT [158]. Elevated LBP levels in cirrhosis were related to proinflammatory state and hemodynamic derangement, which were shown to be ameliorated by intestinal decontamination with norfloxacin [175]. Recently, the detection of bacterial DNA (bactDNA) by polymerase chain reaction has been proposed as a surrogate marker for BT. It has been simultaneously detected in blood and ascites in nine of 28 cirrhotics with culture-negative ascites [176]. The detection of bactDNA in biological fluids in experimental cirrhosis and ascites is associated with its simultaneous presence in mesenteric lymph nodes [177], although uniformity of analytical methods is needed to ascertain its real value in the clinical setting [158].

## 5. Conclusions

There are individual differences in susceptibility to ALD and NASH. Not all heavy drinkers or obese persons have advanced cirrhosis. Apart from host genomics, host-gut microbiota metabolic interactions may become the key to explain the relative sensitivity to alcohol or high-fat, high-sugar diet. To know the whole story in relation to the gut-liver axis may surely improve the management of liver diseases. The impact of gut microbiota on the host is increasing with the progression of liver injury from mild ALD and NAFLD to advanced cirrhosis. Pathological BT in liver cirrhosis was finely delineated in the review by Wiest *et al.* [178]. There is sufficient evidence to justify a rational attempt to modulate the intestinal microbiome to treat liver disease [6]. The usefulness and the limitations of selective intestinal decontamination should be clearly defined and judicious combinations of probiotics and prebiotics should be explored in cirrhotics [33]. Readers interested in the management of gut microbiota in liver cirrhosis are recommended to read recent reviews [33,158]. The management of ALD and NAFLD by targeting microbiota may become an exciting step to the future of gastroenterology.
